# *Lonicerae Japonicae Flos* extract and chlorogenic acid attenuates high-fat-diet- induced prediabetes *via* CTRPs-AdipoRs-AMPK/PPARα axes

**DOI:** 10.3389/fnut.2022.1007679

**Published:** 2022-10-14

**Authors:** Chengcheng Guo, Xiaoyuan Zhang, Yingxiang Yu, Yifan Wu, Lan Xie, Cuiqing Chang

**Affiliations:** ^1^Department of Sports Medicine, Beijing Key Laboratory of Sports Injuries, Peking University Third Hospital, Beijing, China; ^2^Institute of Sports Medicine, Peking University, Beijing, China; ^3^Department of Endocrinology and Metabolism, Peking University People’s Hospital, Beijing, China

**Keywords:** prediabetes, lipid metabolism, chlorogenic acid, *Lonicerae Japonicae Flos* extract, adiponectin receptors, ELOVL6

## Abstract

Prediabetes is considered an important reversible checkpoint in T2DM development, which can be delayed and prevented by early interventions. *Lonicerae Japonicae Flos* (LJF), an edible-medicinal herb, is rich in chlorogenic acid (CGA, 5-O-caffeoylquinic acid) and exerts anti-diabetes effects, but its role in prediabetes remains unclear. The purpose of this study was to explore the effects of LJF extract and CGA on rat with prediabetes. Sprague-Dawley rats were given high-fat diet (HFD) to induce prediabetes, and glycolipid metabolism parameters and molecular mechanisms were evaluated. LJF (the LJF extract treatment group) and CGA (the pure CGA treatment group) significantly attenuated HFD-induced prediabetes with impaired glucose tolerance and dyslipidemia, but their mechanisms of action are not exactly the same. Specifically, LJF prioritizes increasing protective lipid species [such as increasing blood polyunsaturated fatty acids (PUFA)-containing diacylglycerol (DAG) species, high-density lipoprotein-cholesterol (HDL-C)], whereas CGA prioritizes reducing detrimental lipid species [such as saturated fatty acid-containing DAG species, low-density lipoprotein-cholesterol (LDL-C), total cholesterol (TC)]. In addition, CGA significantly increased the content of blood very-long-chain fatty-acid (VLCFA)-containing ceramides species. This could be explained mechanically by a distinction between LJF and CGA’s effects on C1q/TNF-related proteins (CTRPs) which activate adiponectin receptors, triggering several downstream reactions. Because both LJF and CGA upregulated liver expression of adiponectin receptors (AdipoR1 and AdipoR2) and enhanced the activity of downstream AMPK. LJF also increased serum levels of CTRP3 and CTRP9, especially CTRP9, whereas CGA had higher serum CTRP3 and upregulated liver PPARa expression. Additionally, ELOVL6 expression in the liver was greater in CGA than LJF. This study demonstrates that LJF and CGA exert hypoglycemic and lipid modulation capacity to prevent prediabetes may through the CTRPs-AdipoRs-AMPK/PPARα axes and promoting ELOVL6 protein expression.

## Introduction

Prediabetes, a state of neither normoglycemia nor bona-fide diabetes (1, 2), is identified by laboratory measurement of fasting blood glucose (FBG), 2-h postprandial blood glucose (2hBG), or glycosylated hemoglobin (HbA1C) (3). The increasing prevalence of prediabetes globally is a major public health concern and is also associated with an increased risk of type 2 diabetes (T2D) and cardiovascular diseases (4, 5). Diabetes prevention guidelines currently recommend lifestyle modification (dietary changes and increased physical activity) as the first line treatment option for individuals with prediabetes, a critical reversible stage of T2D (6). However, the challenge is sustaining lifestyle change in practice, and even after lifestyle intervention and metformin therapy, a proportion of prediabetes patients progress to T2D (7). Therefore, finding effective and accessible methods to prevent prediabetes from progressing to T2D is crucial.

Plant extracts rich in phenols have been increasingly recognized for their hypoglycemic effects and ability to regulate glucose metabolism in the body, as well as their ease of availability, low cost, and minimal side effects (8). *Lonicerae Japonicae Flos* (LJF) is a polyphenol-rich traditional medicinal herb widely cultivated in eastern Asia. LJF extracts have been explored for treating T2D and its complications, including anti-hyperglycemic, hypolipidemic, and attenuating diabetic retinopathy (9, 10). Additionally, LJF extracts combined with metformin increase metformin distribution in the liver, and ameliorate glucose tolerance (11, 12). The medicinal parts of LJF are derived from the dried buds and initial flowers, and its major active ingredient is chlorogenic acid (5-caffeoylquinic acid, CGA) (13). Previous studies reveal that CGA plays important and therapeutic roles in anti-inflammatory, anti-oxidant, anti-obesity (14), and antidiabetic (15–17). Furthermore, our previous study found that CGA (99% purity, extracted from green coffee seeds) decreases the expression of hepatic glucose-6-phosphatase, and increases adiponectin receptors (AdipoRs), adiponectin, and phosphorylation of AMPK and PPARα in late diabetic mice (18). However, there is little information available about how LJF extracts and its primary active ingredient CGA influence prediabetes, nor is the exact mechanism understood.

Clinical and experimental studies have suggested that C1q/TNF-related protein (CTRP) superfamily (including adiponectin, CTRP3, CTRP6, and CTRP9) bind to their receptors AdipoR1 and AdipoR2, could ameliorate impaired glucose tolerance, insulin resistance (IR) *via* activation of AMPK and PPAR-α pathways, respectively (19, 20). Of these, AdipoR1 [identified as the receptor for adiponectin, CTRP6 (21), and CTRP9 (22, 23)] has been shown to activate the AMP-activated protein kinase (AMPK) pathway, and AdipoR2 (identified as the receptor for adiponectin and CTRP3) (24) to activate peroxisome proliferator-activated receptor (PPAR) signaling, thus improving glucose and lipid metabolism and exerting anti-diabetic effects. In addition, the crystal structure of the AdipoRs suggests that AdipoR1 and AdipoR2 possess intrinsic ceramidase activity (25) and promotes desaturase activity (26). Thus, we hypothesized that LJF extracts and its primary active ingredient CGA ameliorate prediabetes through CTRPs- AdipoRs-AMPK/PPARα Axes.

Here, HFD induced prediabetes rats were established as an experimental animal model to evaluate the effects and the mechanism of LJF extract and CGA on the improvement of glucose and lipid metabolism.

## Materials and methods

### *Lonicerae Japonicae Flos* extract

*Lonicerae Japonicae Flos* bud extract (measured 52.52 % purity CGA, abbreviated as LJF), and CGA (extracted from *Lonicerae Japonicae Flos* bud, measured 98.63% purity CGA, purchased from Herbaltone Bio-technology (Shandong, China). The purity of CGA was measured using standard high-performance liquid chromatography (HPLC), a Hypersil Gold C18 analytical column (5 μm, 250 × 4.6 mm). The mobile phase consisted of acetonitrile (A) and water containing 0.2% phosphoric acid (B). The flow rate was 1.0 mL/min, the injection volume was 10 μL and the detection wave length was 327 nm.

### Animals and experimental treatment

All animal protocols were approved by the Animal Care and Use Committee of Peking University (No. LA2017002). Sprague–Dawley (SD) rats (5-week-old, male, *n* = 32) were obtained from Vital River Laboratory Animal Technology Co. Ltd (Beijing, China) and housed two animals per cage in a standard specific-pathogen-free environment, with *ad libitum* access to water and food. After 7 days of acclimatization, rats (*n* = 24) were fed a high-fat diet (HFD; 45% of energy from fat, 20% from protein, and 35% from carbohydrate, with energy density 4.7 Kcal/g; Research Diets, Cat# D12451) for 30 days to induce prediabetes, then randomly distributed into the following experimental groups (*n* = 8 animals/group) received their respective treatments daily by gavage while continuing exposure to high-fat diet for the next 60 days: HFD group, receiving the vehicle (phosphate buffered saline); HFD+LJF group, receiving LJF (80 mg/kg/day); HFD+CGA group: receiving CGA (80 mg/kg/day), positive medicinal control group. Control group (*n* = 8), receiving standard diet throughout the experiment (CON; 13% of energy from fat, 24% from protein, and 63% from carbohydrate, with energy density 3.4 Kcal/g; Keao Xieli Feed) and the vehicle daily by gavage at the second month.

### Sample collection and laboratory measurements

Food intake and body weight were recorded weekly. At the end of the experiment, rats were fasted overnight and sacrificed under 2% sodium pentobarbital (0.3 mL/100 g body weight) anesthesia. Body weight and length (distance from the tip of the nose to the anus) were measured, and Lee’s index calculated


(1) $$L\,e\,e^{\prime }\,s\,i\,n\,d\,e\,x=\sqrt[{3}]{b\,o\,d\,y\,w\,e\,i\,g\,h\,t\,\left(g\right)\times 1000/b\,o\,d\,y\,l\,e\,n\,g\,t\,h\,\left(c\,m\right)}$$

Blood was collected *via* femoral artery puncture and allowed to clot at room temperature for 30 min. Serum was isolated *via* centrifugation at 1,000 × *g* for 20 min, at 4°C, and then stored at –80°C, for further biochemical analyses. Next, pancreas, liver, white adipose tissues in perirenal and epididymal were removed, weighed, and then either snap-frozen in liquid nitrogen, or fixed in 10% formalin, in preparation for later analysis. Visceral fat percentage (VF%) was calculated as the weight (g) of perirenal and epididymal adipose tissues divided by whole body weight (g), then multiplied by 100%.


(2) $$V\,F\%=\frac{\begin{array}{c} t\,h\,e\,p\,e\,r\,i\,r\,e\,n\,a\,l\,a\,n\,d\,e\,p\,i\,d\,i\,d\,y\,m\,a\,l\,a\,d\,i\,p\,o\,s\,e\\ t\,i\,s\,s\,u\,e\,s\,w\,e\,i\,g\,h\,t\,\left(g\right) \end{array}}{w\,h\,o\,l\,e\,b\,o\,d\,y\,w\,e\,i\,g\,h\,t\,\left(g\right)}\times 100\%$$

### Histology

To assess overall tissue morphology, sections (5 μm) of formalin-fixed pancreas was stained with H&E and imaged under a light microscope (CX21; Olympus, Tokyo, Japan). For histopathological scoring, H&E-stained slides were observed by two blinded pathologists. The number of pancreatic islets was determined using x10 magnification.

### Serum biochemistry

Serum triglyceride (TG), total cholesterol (TC), low-density lipoprotein-cholesterol (LDL-C), and high-density lipoprotein cholesterol (HDL-C) concentrations, as well as liver TG and TC concentrations, were measured using commercially available kits (Nanjing Jiancheng Bioengineering Institute, Nanjing, China). Serum adiponectin (Abcam, Cat. # ab108784), C1q tumor necrosis factor-related protein 3 (CTRP3) (J&L Biological, Cat.# JL46562), CTRP6 (J&L Biological, Cat. # JL46565), and CTRP9 (J&L Biological, Cat.# JL46568) concentrations were measured using ELISA kits.

### Oral glucose tolerance test and homeostasis model assessment of insulin resistance

The oral glucose tolerance test (OGTT) was performed three times: at the beginning, after 4 weeks, and after 8 weeks of CGA treatment, respectively. Rats have fasted for 16 h overnight and body weights were measured. Fasting blood glucose levels were read using an ACCU-CHEK Advantage glucometer (Roche, DE, Switzerland) *via* the tail vein. A 50% glucose solution was administered *via* gavage at 2 g/kg body weight, followed by blood glucose readings at 15, 30, 60, and 120 min post glucose challenge. On the last OGTT, insulin levels were assessed in retrobulbar plexus blood samples at 0, 30, 60, and 120 min, after the glucose challenge. The area under the curve of glucose (AUC-G) and insulin (AUC-I) was estimated using the trapezoidal rule. Quantitative estimation of serum insulin was performed using a rat insulin ELISA kit (Millipore, EZRMI-13K, USA), according to the manufacturer’s protocol. Insulin resistance estimation was performed using the homeostasis model assessment method (HOMA-IR), and was calculated using the following formula: HOMA-IR = fasting blood glucose (FBG, mmol/L) × fasting insulin (FINS, μU/L)/22.5.

### Western blot

Liver tissues were homogenized in RIPA buffer and centrifuged at 12,000 × *g* for 20 min. Membranes were incubated with primary antibodies against phospho-IRS1 (Ser307) (p-IRS1) (CST, #2381), total-IRS1 (t-IRS1) (CST, #2382), phospho-Akt (Ser473) (p-Akt) (CST, #4060), total-Akt (t-Akt) (CST, # 4685), AdipoR1 (ab126611), AdipoR2 (ab77612), ELOVL6 (ab69857), AMPK (CST, #5832), p-AMPK (Thr172) (CST, #2535), and peroxisome proliferator-activated receptor-alpha (PPAR-α) (ab24509), and β-actin (C1313). Quantification of chemiluminescent signals was performed using Image J software.

### Lipidomics

The plasma lipidomic profiling analysis was conducted using an UPLC-Q Exactive Orbitrap-MS instrument (Thermo, CA). Briefly, 400 μL of dichloromethane/methanol (volume ratio = 2:1) was added to 100 μL of plasma, with the lipid-containing liquid of the lower layer then collected and dried using nitrogen to obtain the lipid samples. Reverse-phase chromatography was used to separate lipid samples *via* a Cortecs C18 column (2.1 × 100 mm, Waters). Data were acquired using a Q Exactive orbitrap mass spectrometer (MS) (Thermo, CA), combined with the UHPLC system Ultimate 3000 (Thermo, CA). Lipids were identified and quantified using LipidSearch software v4.1.16 (Thermo, CA). Detailed conditions of the LC gradient and MS instrument parameters were the same as in previous literature (27).

Lipids are abbreviated as follows: sterol lipids cholesteryl ester (ChE), sitosterol ester (SiE); sphingolipids ceramide (Cer), dihydroceramide (DHCer), phytoceramide (PhyCer), hexa-ceramides (Hex1Cer), sphingomyelin (SM); glycerolipids triacylglycerol (TAG) and diacylglycerol (DAG); glycerophospholipids phosphatidylethanolamine (PE), phosphatidylinositol (PI). The side-chain structures are denoted as carbon chain length: the number of double bonds and are provided for each chain where they could be determined, or as a total number of all carbons and double bonds where individual chains could not be determined.

### Statistical analysis

All data are presented as mean ± SEM, except where otherwise indicated. SPSS statistics 25 (IBM, Armonk, NY, USA) was used for statistical analysis. Statistical significance was determined using unpaired Student’s *t*-test when analyzing CON group and HFD group, for normally distributed data, and the Mann–Whitney rank sum test, f or non-normally distributed data. Comparisons among three groups (HFD, HFD+LJF, and HFD+CGA) were performed using one-way analysis of variance (ANOVA) followed by Tukey’s postdoc test, for normally distributed data, and Krus-kal-Wallis nonparametric ANOVA with Bonferroni correction, for non-normally dis-tributed data. *P*-values < 0.05 were considered significant.

## Results

### Effect of *Lonicerae Japonicae Flos* and chlorogenic acid on body weight

**FIGURE 1:**
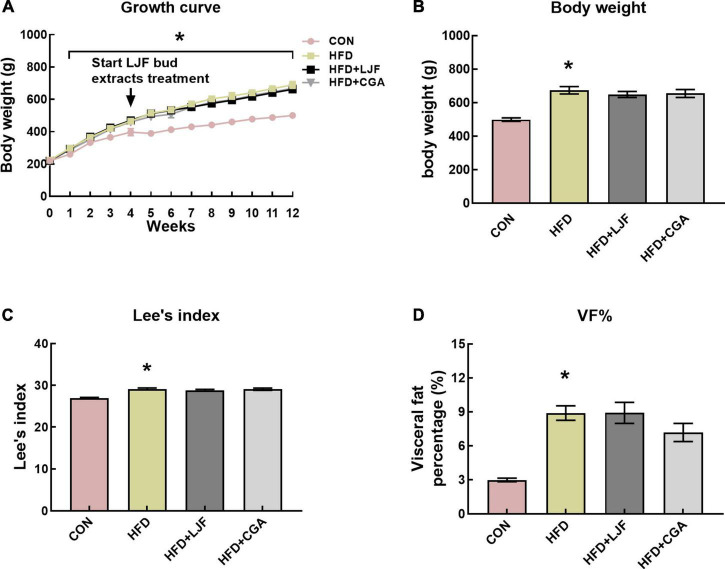
*Lonicerae Japonicae Flos* (LJF) and chlorogenic acid (CGA) have no significant effect on body weight and VF% in prediabetes rats. **(A)** Graph showing evolution of body weight in male SD rats fed a standard diet vs. HFD diet for up to 12 weeks. **(B–D)** Body weights, Lee’s index, and VF% (visceral fat mass as a percentage of body weight) at the end of the study. Data are expressed as means ± SEM (*n* = 8 per group). *P < 0.05, HFD group vs. CON group.

To examine the effects of LJF and CGA on the development of prediabetes, body weight was tracked weekly for 12 weeks (Figure 1A). At the end of the experiment, rats in the HFD group had significantly increased in body weight, Lee’s index, and VF% compared to CON rats (Figures 1B–D). The addition of LJF or CGA did not significantly affect the body weight, Lee’s index and VF% compared to the HFD group (Figure 1D).

### Effect of *Lonicerae Japonicae Flos* and chlorogenic acid on glucose tolerance and insulin sensitivity

#### Effect of *Lonicerae Japonicae Flos* and chlorogenic acid on impaired glucose tolerance

**FIGURE 2:**
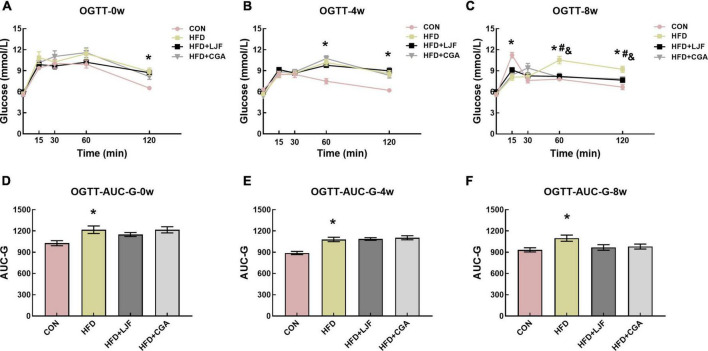
*Lonicerae Japonicae Flos* and CGA protect against HFD-induced IR and glucose homeostasis disorder in prediabetes rats. **(A–C)** Blood glucose during the oral glucose tolerance test (OGTT) was measured at the indicated time points after administration of 2 g/kg glucose to rats, following consumption of PBS, LJF or CGA for 0, 4, or 8 weeks (*n* = 8 per group). **(D–F)** The area under the curve of glucose (AUC-G). Dates are presented as bar graphs with means ± SEM. *P < 0.05, HFD group vs. CON group; ^#^*P* < 0.05, HFD+LJF vs. HFD group; ^&^*P* < 0.05, HFD+CGA vs. HFD group.

To test markers of insulin sensitivity, OGTT was performed on live rats every 4 weeks. At the beginning of LJF and CGA treatment, HFD rats had high blood glucose levels (Figure 2A), at 120 min, and an increased AUC-G (Figure 2D), compared to the CON group. After 4 weeks of LJF and CGA treatment, HFD rats had high blood glucose levels, at 60 and 120 min (Figure 2B), and an increased AUC-G (Figure 2E), compared to the CON group, and this trend continued until the end of the experiment (Figures 2C,F). As shown in Figure 2, impaired glucose tolerance (IGT) in HFD group not only lasted for a further 8 weeks following 4-week-HFD exposure but also progressively worsened. In prediabetes rats fed either LJF or CGA for 4 weeks, OGTT-60 min, OGTT-120 min glucose levels and AUC-G in the LJF group and CGA group did not differ significantly from those in the HFD group (Figures 2B,E). The OGTT-60min and OGTT-120min blood glucose levels of the LJF group and the CGA group decreased significantly after 8 weeks of LJF and CGA treatment, but AUC-G did not (Figures 2C,F).

#### *Lonicerae Japonicae Flos* and chlorogenic acid decreased the level of serum insulin and insulin resistance

**FIGURE 3:**
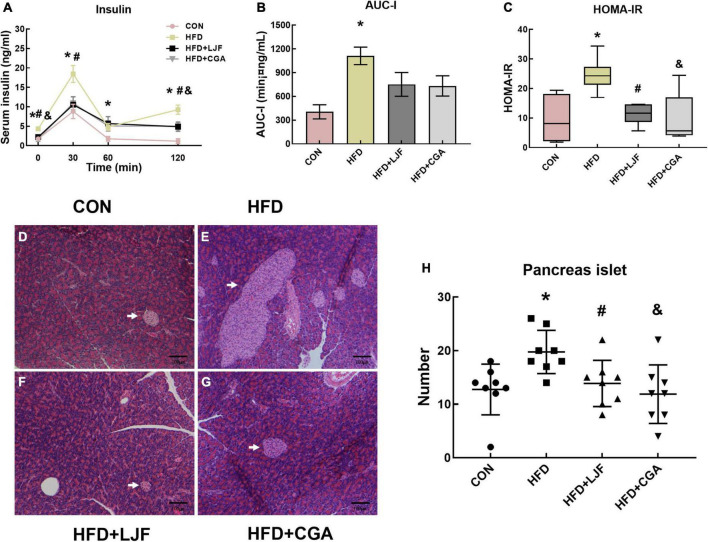
*Lonicerae Japonicae Flos* and CGA protect against HFD-induced IR and glucose homeostasis disorder and significantly reduce islet hyperplasia in prediabetes rats. **(A)** Serum insulin levels during the last OGTT timepoint (*n* = 6 per group). **(B)** The area under the curve of serum insulin (AUC-I) (*n* = 6 per group). **(C)** Homeostasis model assessment of insulin resistance (HOMA-IR) presented as box and whiskers plots, minimum to maximum, where the center line represents the median (*n* = 6 per group). **(D–G)** Representative H&E stained images of the pancreas (Scale bar represents 100 μm; x100) in the indicated group; islets (white arrows). **(H)** Scatter plot graphs (bottom panels) represent the number of pancreatic islets, each dot represents one rat. Other data are presented as bar graphs with means ± SEM. *P < 0.05, HFD group vs. CON group; ^#^*P* < 0.05, HFD+LJF vs. HFD group; ^&^*P* < 0.05, HFD + CGA vs. HFD group.

We next examined serum insulin levels. Our data indicated that rats fed either LJF or CGA for 8 weeks showed better insulin sensitivity. A significant increase in serum insulin was observed in HFD rats at 0, 30, 60, and 120 min compared with CON rats. LJF treated rats had significantly lower insulin levels at 0, 30, and 120 min than HFD treated rats, whereas CGA treated rats had significantly lower insulin levels at 0 and 120 min than HFD rats (Figure 3A). Although AUC-I for rats in both LJF and CGA groups did not differ, compared with the HFD group (Figure 3B), the HOMA-IR index was substantially reduced in both the LJF and CGA groups (Figure 3C). Those results are consistent with pathological findings that rats in CON group had significantly fewer pancreatic islets than rats in HFD group, and rats treated for 8 weeks with LJF or CGA also had significantly fewer pancreatic islets than rats in HFD group (Figures 3D–H). Taken together, although both LJF and CGA do not affect body mass, they still improve insulin sensitivity and glucose homeostasis.

### *Lonicerae Japonicae Flos* and chlorogenic acid improved insulin signaling pathway in liver

**FIGURE 4:**
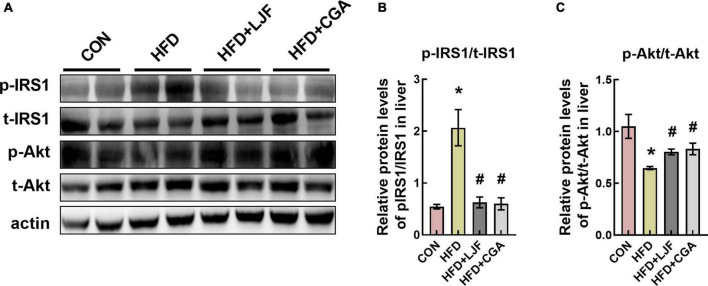
*Lonicerae Japonicae Flos* and CGA improved the insulin signaling pathway in rat liver. **(A–C)** Protein levels and densitometric quantification of phospho-insulin receptor substrate 1 (p-IRS1) (Ser307), total(t)-IRS1, phospho-protein kinase B (p-Akt) (Ser473), total(t)-Akt, and β-actin, bars represent densitometric quantification normalized to β-actin. All bars show means ± SEM. (*n* = 4 per group). *P < 0.05, vs. CON group; ^#^*P* < 0.05, vs. HFD group.

To further assess the effects of LJF and CGA on glucose tolerance, we measured the protein levels of phospho-IRS1(Ser307) and phospho-Akt (Ser473) in rat liver. We found that a HFD significantly increased phosphorylation at Ser307-(p-IRS1), which was accompanied by decreased Akt phosphorylation at Ser473-(p-Akt) in liver. In contrast, the insulin signaling pathway was dramatically ameliorated in both LJF and CGA groups, characterized by reduced IRS-1 (Ser307) phosphorylation and elevated Akt phosphorylation (Figures 4A–C).

### *Lonicerae Japonicae Flos* and chlorogenic acid improved lipid homeostasis

Prediabetes is ultimately accompanied by abnormal lipid metabolism and adipokine disorder. As expected, compared with the CON group, dyslipidemia was present in HFD-induced prediabetes rats, as indicated by increased serum LDL-C and TG (Table 1), as well as increased liver TC and TG content. Conversely, HDL-C levels were significantly decreased than in CON group (Table 1). Compared with the HFD group, we found that LJF treated rats had significantly higher serum HDL-C, while CGA treated rats had significantly lower hepatic TC and serum LDL-C levels (Table 1).

**TABLE 1:** Effect of *Lonicerae Japonicae Flos* (LJF) and chlorogenic acid (CGA) on blood lipid and liver lipid content.

Group	CON	HFD	HFD + LJF	HFD + CGA
N	8	8	8	8
HDL-C (mmol/L)	0.38 ± 0.04	0.31 ± 0.06*	0.51 ± 0.23^#^	0.33 ± 0.14^&^
LDL-C (mmol/L)	0.79 ± 0.11	1.13 ± 0.26*	1.01 ± 0.27	0.78 ± 0.09^#^
TG (mmol/L)	0.56 ± 0.08	0.66 ± 0.08*	0.90 ± 0.50	0.78 ± 0.26
TC (mmol/L)	2.17 ± 0.37	2.15 ± 0.44	2.31 ± 0.66	2.01 ± 0.22
TG-Liver (mmol/mg)	0.10 ± 0.04	0.21 ± 0.10*	0.15 ± 0.06	0.17 ± 0.06
TC-Liver (mmol/mg)	0.09 ± 0.05	0.19 ± 0.10*	0.14 ± 0.02	0.12 ± 0.06^#^

Serum HDL-C (high-density lipoprotein cholesterol), LDL-C (low-density lipoprotein cholesterol), TC (total cholesterol) and TG (triglyceride) levels, and liver TC and triglyceride (TG) content in rats. Data are expressed as mean ± SD. *P < 0.05, vs. CON group; ^#^*P* < 0.05, vs. HFD group; ^&^*P* < 0.05, vs. HFD+LJF. mmol/mg refers to the content of TG or TC per milligram of protein in the liver.

**FIGURE 5:**
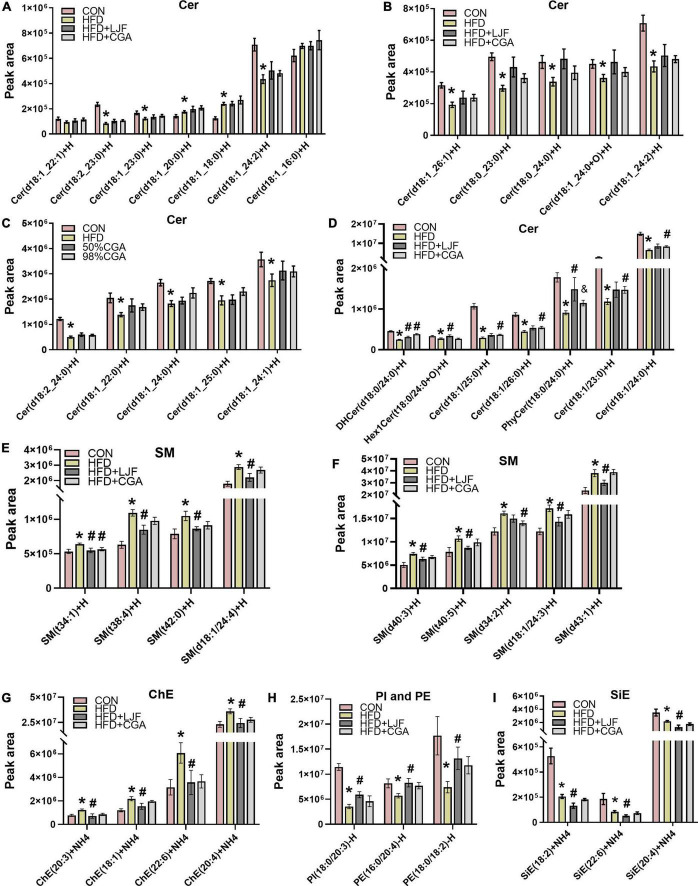
*Lonicerae Japonicae Flos* and CGA regulated plasma sphingolipids levels. **(A–D)** Lipidomic Analysis in rat plasma concentrations of ceramide (Cer) species. **(E–I)** Plasma concentrations of sphingomyelin (SM), cholesteryl ester (ChE), sitosterol ester (SiE), phosphatidylethanolamine (PE), and phospha-tidylinositol (PI). Data are presented as bar graphs with means ± SEM (*n* = 8 per group). *P < 0.05, vs. CON group; ^#^*P* < 0.05, vs. HFD group; ^&^*P* < 0.05, vs. HFD + LJF.

Next, we performed lipidomic analysis due to the high complexity of the plasma lipidome. At the end of the experiment, the HFD diet significantly elevated plasma levels of Cer (d18:1/18:0), with neither LJF nor CGA attenuating this increase, However, the Cer (d18:1/18:0) levels showed a small but not significant increase in the CGA group than in the LJF group. By contrast, the HFD diet significantly reduced the contents of many very long chain-containing ceramides species (Figures 5A–D), some of which can be reversed by LJF and CGA treatment. In detail, CGA significantly attenuating the reduction of Cer (d18:1/23:0), Cer (d18:1/24:0), Cer (d18:1/25:0) and Cer (d18:1/26:0) (Figure 5D). However, Hex1Cer (t18:0/24:0+O) and PhyCer (t18:0/24:0) (Figure 5D) contents were reduced in the HFD group with LJF attenuating the reduction, and similar trends were observed for the PI and PE species (Figure 5H). Furthermore, the level of DHCer (d18:0/24:0) reduced in the HFD group was significantly increased by both LJF and CGA (Figure 5D). By contrast, the amount of SM and ChE species combined was increased by the HFD diet, with LJF significantly attenuating this increase whereas CGA supplementation did not affect those species (Figures 5E–G). Interestingly, LJF exerted specific effects on several SiE species that were decreased by the HFD diet, such as the reduction of SiE (18:2), SiE (20:4), and SiE (22:6) levels (Figure 5I). For more details, see Supplementary material.

**FIGURE 6:**
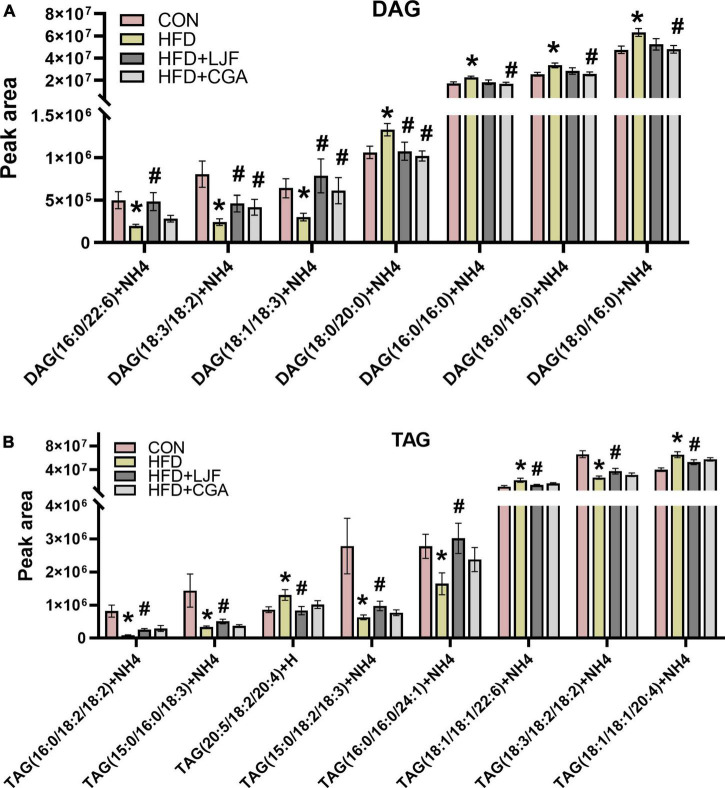
*Lonicerae Japonicae Flos* and CGA regulated plasma diacylglycerol (DAG) and triacylglycerol (TAG) species levels. Lipidomic Analysis in Rat **(A)** plasma concentrations of DAG species. **(B)** Plasma concentrations of TAG species. Data are presented as bar graphs with means ± SEM (*n* = 8 per group). *P < 0.05, vs. CON group; ^#^*P* < 0.05, vs. HFD group; ^&^*P* < 0.05, vs. HFD + LJF.

We have also analyzed the fatty acid profile of the plasma TAGs and DAG. Notably, levels of C18:2, C18:3, and C24:1 containing-TAGs were significantly reduced in the HFD group with LJF attenuating this change. The opposite trend is observed in concentrations of C18:1 and C20:4 containing-TAGs such as TAG (20:5/18:2/20:4), TAG (18:1/18:1/20:4), and TAG (18:1/18:1/22:6), whereas CGA treatment not reversing those changes (Figure 6B). As there were for DAG species, there were decreases in the abundance of C18:2, C18:3, and C22:6 containing DAG species with both LJF and CGA strikingly reversing this decrease, excepting DAG (16:0/22:6) (LJF only). However, the levels of DAG species containing C16:0 and C18:0, including DAG (16:0/16:0), DAG (18:0/18:0), and DAG (18:0/16:0), elevated in HFD animals were decreased in the CGA group only, whereas elevated DAG (18:0/20:0) in HFD reversed by both LJF and CGA treatment (Figure 6A).

### *Lonicerae Japonicae Flos* and chlorogenic acid activate the adiponectin pathway

To gain more insight into the mechanisms involved in the regulation of IGT and lipid metabolism, we examined the effects of LJF and CGA treatment on CTRPs-AdipoRs signaling pathways and ELOVL6 which regulates the acyl-chain composition of ceramide (28).

Results showed that lower serum adiponectin, CTRP3, and CTRP9 levels of serum were observed in rats exposed to HFD feeding when compared with CON group. Compared with HFD group, the level of serum CTRP3 was higher following LJF and CGA treatment, with CTRP3 significantly higher in the CGA group than in the LJF group. In addition, decreased serum CRTP9 in HFD-fed rats was increased by LJF. These results indicate that both LJF and CGA have therapeutic effects on the reversal of adipokine disorder (Table 2).

**TABLE 2:** *Lonicerae Japonicae Flos* and CGA improved serum adipokine disorder.

Group	Adiponectin (ug/mL)	CTRP3 (ng/mL)	CTRP6 (ng/mL)	CTRP9 (ng/mL)
CON	5.52 ± 1.55	179.85 ± 29.90	72.48 ± 9.76	27.79 ± 9.77
HFD	3.94 ± 0.98*	133.52 ± 8.06*	79.67 ± 10.49	11.27 ± 1.84*
HFD+LJF	3.94 ± 0.73	148.75 ± 9.77^#^	74.29 ± 7.72	18.30 ± 1.88^#^
HFD+CGA	3.51 ± 0.54	181.64 ± 17.11^#&^	79.49 ± 9.57	12.12 ± 1.42^&^

Circulating levels of Adiponectin, CTRP3, CTRP6, and CTRP9 in prediabetes rats. CTRP3, CTRP6, and CTRP9, C1q tumor necrosis factor related protein 3, 6, and 9. Data are expressed as mean ± SD. *P < 0.05, vs. CON group; ^#^*P* < 0.05, vs. HFD group; ^&^*P* < 0.05, vs. HFD+LJF. Data are presented as bar graphs with means ± SD (n = 8 per group).

Compared with the CON group, the liver expression level of AdipoR1 and AdipoR2 expression did not change significantly in the HFD group. Interestingly, rats treated with LJF or CGA for 8 weeks showed significantly upregulated expression levels of AdipoR1 and AdipoR2 than the HFD group. Next, we analyzed PPARα expression and the phosphorylation of AMPK, because AdipoR1 activates the AMPK pathway and AdipoR2 activates the PPARα pathway, respectively (29). We found that the phosphorylation of AMPK was significantly increased by LJF and CGA administration. And the CGA administration markedly increased the expression of PPARα which was decreased in HFD group. These results suggest that LJF enhances lipid metabolism partially by the regulation of the CTRP9/ AdipoR1/AMPK axis, while CGA *via* the CTRP3/AdipoR2/PPARa axis.

**FIGURE 7:**
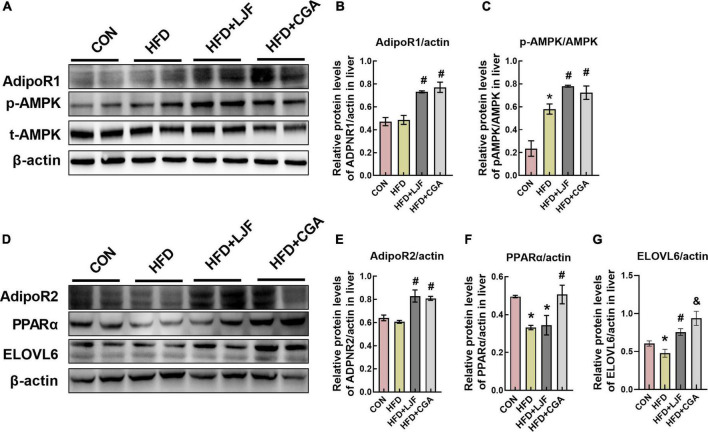
*Lonicerae Japonicae Flos* and CGA up-regulated expression levels of AdipoR1, AdipoR2, and ELOVL6 proteins in rat liver. **(A–G)** Protein levels and densitometric quantification of adiponectin receptors (AdipoR1 and AdipoR2), ELOVL6, p-AMPK, AMPK, PPAR-α, and β-actin, bars represent densitometric quantification normalized to β-actin. All bars show means ± SEM. (*n* = 4 per group). *P < 0.05, vs. CON group; ^#^*P* < 0.05, vs. HFD group; ^&^*P* < 0.05, vs. HFD + LJF.

Furthermore, the liver ELOVL6 expression was also reduced in the HFD group versus the CON group, and both LJF and CGA attenuated this reduction. Interestingly, the hepatic ELOVL6 level was significantly higher in the CGA group than in the LJF group (Figure 7).

## Discussion

Prediabetes is strongly associated with a high risk of T2D and is an important reversible checkpoint in T2D development. In this investigation, both LJF and CGA improved HFD-induced prediabetes with IGT and lipid metabolism disorders such as decreasing plasma LDL-C and liver TC, attenuating the reduction of plasma HDL-C, VLCFA-containing ceramides species and PUFA-containing DAG species, which may be through the CTRPs-AdipoR1/2-AMPK/PPARα signaling pathway. In detail, LJF has a stronger effect on the CTRP9-AdipoR1-AMPK axis, while CGA has a stronger effect on the CTRP3-AdipoR2-PPARa axis. In addition, ELOVL6 protein was also involved in regulating the chain length of ceramide.

Administration of the HFD diet for 2 months caused an increase in body weight and adiposity, disturbance in lipids and adipokines, impaired glucose tolerance, β-cell mass expansion, and hyperinsulinemia compared to control rats, but not FPG. This suggests that impaired glucose tolerance and hyperinsulinemia induced by the HFD diet occur in an early stage of prediabetes. Although neither LJF nor CGA intervention impacted body weight, both of them enhanced glucose tolerance as evidenced by reduced blood glucose levels at 60 and 120 min during the OGTT and HOMA-IR, which is consistent with the decreased phosphorylation of IRS1(Ser307) and increased phosphorylation of Akt (Ser473) in liver. PPARα and AMPK are major regulators of glucose and lipid metabolism. The phosphorylation of AMPK improves hepatic IRS1-PI3K-AKT signaling pathway and increases hepatic insulin sensitivity (30, 31). Moreover, AdipoR1 demonstrated as the receptor for CTRP9 (22, 23) has been shown to activate the AMPK pathway, whereas AdipoR2 is identified as the receptor for CTRP3 (24) to activate PPARα pathway. In this study, both LJF and CGA increased the expression of AdipoR1 and AdipoR2, and their downstream phosphorylation of AMPK, which may partially account for the beneficial effects of LJF and CGA on lipid and glucose metabolism. Interestingly, the CGA group has a higher plasma CTRP3 than LJF, which may help to increase PPARα expression and decrease the level of plasma LDL-C and liver TC, which were not observed in LJF. Thus, these findings suggest that the CGA group has stronger effect on the CTRP3-AdipoR2-PPARα axis than the LJF group. Accordingly, we speculate that CGA promoted CTRP3 and PPARα expression may be in a dose-dependent manner. Additionally, CTRP9 increased only in LJF group indicating a stronger effect on the CTRP9-AdipoR1-AMPK axis than in the CGA group.

Notably, the plasma lipidomic analysis in our study also offered some clues to explain these positive features of LJF and CGA supplements. Several studies have proved that sphingolipids are signal molecules that interfere with insulin signaling, while ceramides are one of the most important bioactive lipids playing different roles in the development of IR depending on the length of the acyl chain (32). For instance, obesity usually causes increased concentrations of Cer (d18:1/16:0) and Cer (d18:1/18:0), which inhibit the insulin receptor-PI3K-AKT signaling pathway and fatty acid β-oxidation, thus leading to the onset of IR (33). Conversely, Cer (d18:1/24:0), preferentially synthesized by ceramide synthase 2, is suggested to protect mice from HFD-induced obesity and glucose intolerance (34). Additionally, dihydroceramide (DHCer) is transiently produced during the process of ceramides *de novo* synthesis, finally being desaturated by two isoforms of dihydroceramide desaturase (Des1/2) to form ceramides, for example, Cer (d18:1/X). Previous studies have reported that inhibition of Des1 activity may increase DHCer levels, preferentially producing DHCer (d18:0/24:0), thus inducing cytoprotective autophagy (35), and improving glucose tolerance and insulin sensitivity without affecting body mass (36, 37). Phytoceramide (PhyCer), first found in plants and yeast, but also in animal brains, heart, and liver tissue is an intermediate product present during the conversion of DHCer to ceramides (38). A previous study showed that plant-derived PhyCer promotes the PI3K-AKT signaling pathway (39). In agreement with our findings, both LJF and CGA treatments ameliorated the decrease of plasma DHCer (d18:0/24:0) and PhyCer (t18:0/24:0) induced by HFD. Moreover, CGA treatment reversed the reduction of VLCFA-containing Cer (d18:1/C23-26:0).

Mechanistically, ELOVL6 is responsible for converting C12, C14, and C16 saturated and monounsaturated fatty acids into C18 species and regulating the acyl-chain composition of ceramide. Elovl6 is also the major elongase acting on odd-chain SFA C13 and C15 fatty acids, which catalyzed elongation of C13→C15 and C15→C17 (40, 41). Simultaneously, others demonstrated that downregulation of Elovl6 expression with siRNA decreased levels of very long-chain species in ceramides such as Cer (d18:1/C24:0) and Cer(d18:1/C26:0) (42). Furthermore, ELOVL6-KO mice exhibit an impaired metabolic profile, expanded β-cell mass, and increased insulin secretory capacity (43). In Tang et al.’s study, ELOVL6-KO mice gain weight and have increased subcutaneous white adipose tissue mass and impaired carbohydrate metabolism, and had lower brown adipose tissue thermogenic capacity due to the activity of Elovl6 is required for the remodeling of mitochondria for enhancing thermogenic potential (44, 45). Thus, the increased expression of Elovl6 protein may partially explain the common positive performance between LJF and CGA, including elevated DHCer (d18:0/24:0), and Phy (t18:0/24:0), and attenuated β-cell mass expanding. Given that the content of CGA in the CGA group was almost equivalent to twice that of the LJF group, and the CGA group exerts higher expression of ELOVL6 level that explains increasing VLCFA-containing Cer (d18:1/C23-26:0) in the CGA group.

It is well-known that AdipoR1 and especially AdipoR2 possess intrinsic ceramidase activity of lowing ceramide levels. In terms of AdipoR2, which has a preference for Cer (d18:1/18:0) substrate, the ceramidase activity was greatly increased (20-fold) when treated with adiponectin (25, 46) which shared AdipoR1 as a common receptor with CTRP9, and AdipoR2 as a coreceptor with CTRP3. Taken together, we speculate that ELOVL6-induced Cer (d18:1/18:0) lipotoxicity was abolished by the ceramidase activity of the AdipoR1/2 (28), and retained the beneficial effects of ELOVL6 on insulin sensitivity. This may explain that CGA treatment improves insulin sensitivity without reducing Cer (d18:1/18:0) and even a small higher content was shown in the CGA group. These results provide evidence for CGA to exert synergistic action in improving insulin sensitivity *via* a different mechanistic pathway. Moreover, AdipoR1 and AdipoR2 also have been demonstrated to promote FA desaturation and increase the levels of long-chain PUFAs *via* their enzyme activity independently of adiponectin (26), which may account for the effectiveness of lowering the saturated fatty acids content like C18:0 or C16:0-containing DAG and increasing in unsaturated fatty acids like C18:1, C18:2, C18:3, and C22:6-containing DAG accompanied with upregulation of liver AdipoR1 and AdipoR2 expression in both LJF and CGA treatment.

The different effects of LJF compared to CGA were also observed with SM, ChE, PE, and PI species, whose levels were reduced by LJF treatment compared to the HFD group, but not by CGA treatment. Sphingomyelin synthase (SMS), using ceramide as one of the substrates to produce sphingomyelin, has 2 isoforms: SMS1 and SMS2. SMS2 is the major isoform in the liver. However, SM can be hydrolyzed to ceramide by 5 different sphingomyelinases. Importantly, SMS2 deficiency prevented high fat diet-induced obesity and insulin resistance. SMS2 liver-specific knockout mice could diminish liver steatosis (47).

Although TAGs are not signaling molecules, fatty acids produced during their synthesis or breakdown were shown to interfere with the intracellular insulin signaling pathway and contribute to the development of insulin resistance. Previous studies showed positive correlations between levels of C18:2 and C18:3 containing TAGs and insulin sensitivity, whereas levels of saturated fatty acids C20:0 and C18:0 and monounsaturated C18:1 and C20:4 containing TAGs were shown previously to negatively correlate with insulin resistance (48), which according to line with our results. Given that CGA alone did not cause such a reduction, the different effects of LJF compared to CGA might be regarded as a positive effect of other compounds contained in the LJF. A recent study suggests that the main three bioactive components of LJF are organic acids, iridoids and amino acids (49). However, further studies are needed to investigate this in more detail.

One limitation of our study is that we calculated HOMA-IR, an indirect method, to assess β-cell function and insulin sensitivity, rather than using the gold standard hyperinsulinemic euglycemic clamp technique. Although both of these methods have the same sensitivity, HOMA-IR has a lower precision. An additional limitation of our study is that we do not know the identity of the other bioactive compounds in LJF bud extracts, which therefore limits our interpretation of its mechanism of action.

## Conclusion

This study demonstrates that LJF and CGA exert hypoglycemic and lipid modulation capacity to prevent prediabetes in different ways. Specifically, LJF prioritizes raising protective lipid species (such as increasing blood polyunsaturated fatty acids (PUFA)-containing diacylglycerol (DAG) species, high-density lipoprotein-cholesterol (HDL-C)) *via* CTRP3/9-AdipoR1-AMPK pathway, whereas CGA prioritizes reducing detrimental lipid species (such as saturated fatty acid-containing DAG species, low-density lipoprotein-cholesterol (LDL-C), total cholesterol (TC)) *via* CTRP3-AdipoR2- PPARα pathway. This indicated that LJF and CGA as a potential therapy to prevent metabolic disorders in multiple targets. Thus LJF bud extracts rich in CGA could be considered a potential candidate for the prevention and treatment of prediabetes at the earlier stage.

## Data availability statement

The original contributions presented in this study are included in the article/Supplementary material, further inquiries can be directed to the corresponding author.

## Ethics statement

This animal study was reviewed and approved by the Animal Care and Use Committee of Peking University (No. LA2017002).

## Author contributions

CC: conceptualization, funding acquisition, project administration, resources, and supervision. CG: formal analysis and writing original draft. CG, XZ, YY, YW, and LX: investigation. All authors read and agreed to the published version of the manuscript.
