# Supplementary material for: Lonicerae Japonicae Flos extract and chlorogenic acid attenuates high-fat-diet- induced prediabetes via CTRPs-AdipoRs-AMPK/PPARα axes
Source: Front Nutr. 2022 Oct 14;9:1007679. doi: 10.3389/fnut.2022.1007679 (PMC9614216; doi:10.3389/fnut.2022.1007679)
Supplement: Supplementary file 1 [file Data_Sheet_1.zip › Supplementary Material/Notes-lipidomics.docx]

Notes:

Group “50CGA” means: HFD+LJF

Group “98CGA” means: HFD+CGA
